# The extracellular vesicles targeting tumor microenvironment: a promising therapeutic strategy for melanoma

**DOI:** 10.3389/fimmu.2023.1200249

**Published:** 2023-07-28

**Authors:** Yongmin Li, Fei Liu

**Affiliations:** ^1^ Department of Colorectal Surgery, Cancer Hospital of China Medical University, Liaoning Cancer Hospital and Institute, Shenyang, China; ^2^ Department of Bone and Soft Tissue Tumor Surgery, Cancer Hospital of China Medical University, Liaoning Cancer Hospital & Institute, Shenyang, China

**Keywords:** extracellular vesicles, tumor microenvironment, immunotherapy, melanoma, cancer

## Abstract

Extracellular vesicles (EVs) are small particles secreted by numerous cell types and circulate in almost all body fluids, acting as crucial messengers for cell-to-cell communication. EVs involves multiple physiological and pathological processes, including tumor progression, via their multiple cargoes. Therefore, EVs have become attractive candidates for the treatment of tumor, including melanoma. Notably, due to the crucial role of the tumor microenvironment (TME) in promoting tumor malignant phenotype, and the close intercellular communication in TME, EVs-based therapy by targeting TME has become a cutting-edge and prospective strategy for inhibiting melanoma progression and strengthening the anti-tumor immunity. In this review, we aimed to summarize and discuss the role of therapeutic EVs, which target the components of TME in melanoma, thereby providing insights into these promising clinical strategies for the treatment of melanoma patients.

## Introduction

Melanoma is an extremely malignant tumor accounting for approximately 5% of all tumors, which arises from melanocytes (1). Melanoma mainly involves the skin, and can also occur in eyes, meninges and diverse mucosal surfaces (2). Although melanoma is rare, it is responsible for most skin cancer-related death (3), owing to the great metastatic feature of melanoma cells (4). In addition to the tumor-draining sentinel lymph node (SLN) that has been recognized as the most initial metastasis site, melanoma cells also frequently disseminate to the distant regions and organs (5, 6). The tumor microenvironment (TME), a complex environment consists of various cells, including tumor cells, neutrophils, myeloid-derived suppressor cells (MDSC), immune and stromal cells, is vital for the development of melanoma, and exerts a key role in modulating both tumor immunity and the prognosis of melanoma (7–9). Notably, these impacts are mainly mediated by the crosstalk between melanoma tumor cells and other types of TME cells.

In the past decades, the lipid membrane bound nanoparticles extracellular vesicles (EVs), which can be produced by most cells, have been identified as important mediators during the communication between cells (10). EVs have been suggested to be divided into several subtypes according to their size (small and large EVs), origin (exosomes, ectosomes and apoptotic bodies), biochemical components (CD63^+^/CD81^+^ EVs) and physiological condition (hypoxic EVs) (11, 12). Although the classification of EVs is distinct, and it is difficult to distinguish them accurately, recent technological advances enable a more refined differentiation of the subset of EVs by diverse markers (13). EVs contain a great variety of biomolecules released from their donor cells, such as proteins, nucleic acids, and lipids. By transmitting their cargo to adjacent or distant recipient cells or tissues, EVs are able to trigger the malignant phenotype changes of the receptor cell, including the augmented tumor cell migration and invasion, enhanced angiogenesis, and impaired tumor immunity (14, 15).

**Figure 1:**
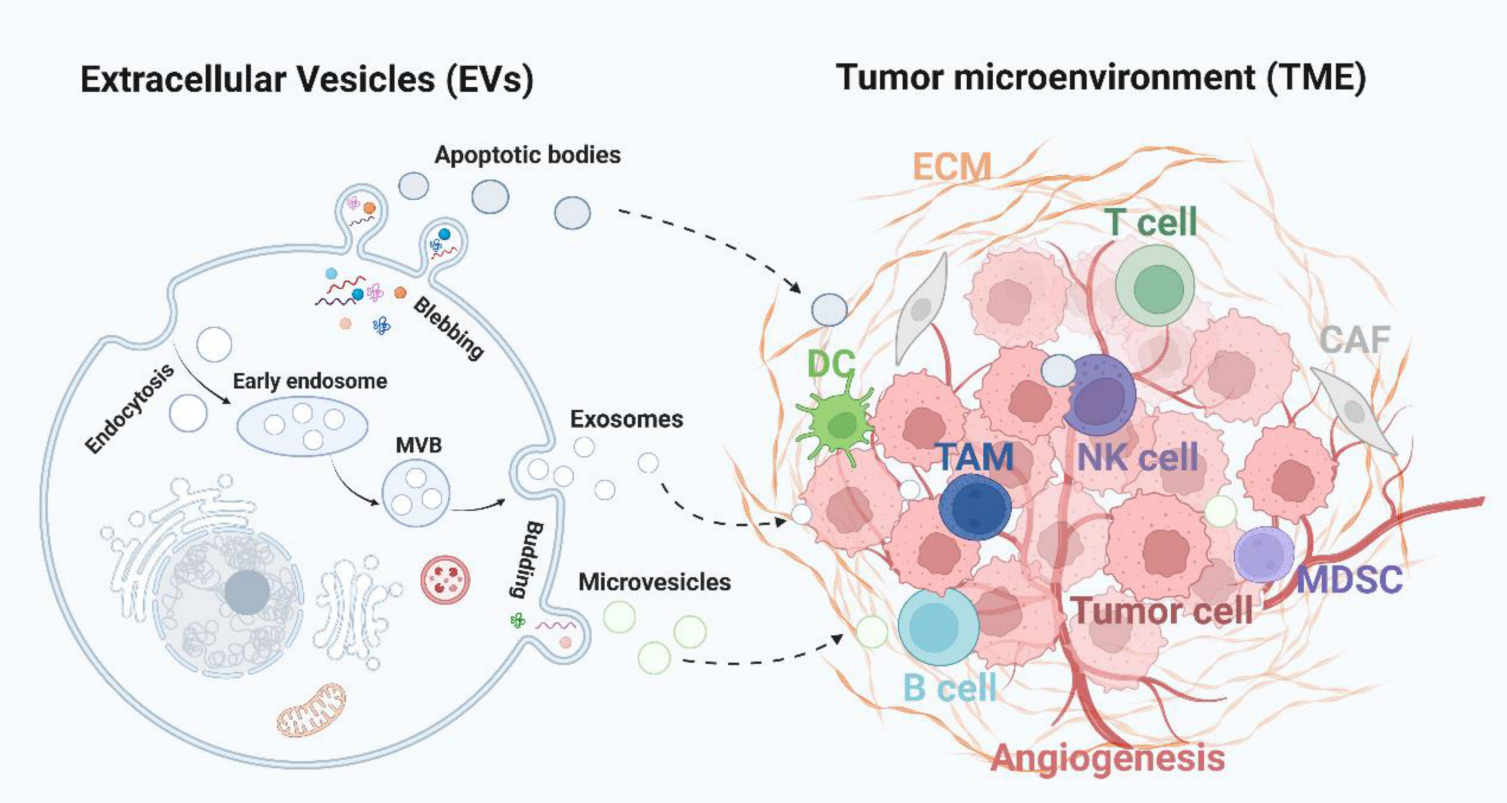
Extracellular vesicles-mediated intercommunication with TME. Tumor cells derived EVs, including exosomes, microvesicles and apoptotic bodies, deliver various cargoes to the TME and influence the immune response (TAM, DC, and T, B, NK cells), angiogenesis, stromal cells activity (CAF and MDSC), and ECM formation. CAF, cancer-associated fibroblasts; ECM, extracellular matrix; MDSC, myeloid-derived suppressor cells; TAM, tumor-associated macrophages; NK cell, natural killer cell.

Recently, increasing evidence have shown the close relationship between EVs and TME (**Figure 1**), which is responsible for the tumor progression, including melanoma (15–17). Therefore, in this review, we aimed to summarize the role of EVs in melanoma by targeting TME, and discuss the potential of EVs to be applied as an alternative option for the clinical treatment of melanoma.

## Crosstalk between EVs and stromal cells in melanoma

In the microenvironment of tumor, stromal cells are vital components and play important roles in the occurrence, development and metastasis of tumor (18, 19). Among them, cancer-associated fibroblasts (CAFs) is the main type of the reactive tumor stroma and contributes to the tumorigenesis, such as melanoma (20, 21). Tumor-related EVs have been demonstrated to be involved in the differentiation of normal fibroblasts (NFs) into CAFs, and then induced a tumor-stimulative stroma (**Table 1**) (29). Gm26809, a novel lncRNA, was upregulated obviously in cytotoxic T-lymphocytes CTLL2 under the stimulation of EVs derived from B16F0 melanoma cells (30). Moreover, Hu et al. (22) found that the EVs released by B16F0 cell can induce the reprogram of fibroblast NIH/3T3 cells to CAFs, as well as the facilitated Cloundman S91 melanoma cell proliferation and migration, which was mediated by delivering Gm26809 to NIH/3T3 cells. These findings suggest that EVs-Gm26809 might be a potential target for promoting the progression of melanoma. Nevertheless, the potential molecular mechanism about how EVs-Gm26809 regulated the transition of NFs to CAFs remains unclear, and whether melanoma-derived EVs could transfer Gm26809 to other stromal cells required further investigation. The miRNAs with 18–24 nucleotide are major members of the non-coding RNAs, which can control the translation of downstream tumor-associated mRNAs, thus participating the tumor progression (31, 32). The miRNAs secreted by melanoma cells have also been implicated in the transformation of NFs towards CAFs by changing CAFs-related genes expression (33, 34). Similar to EVs, melanosomes with a diameter of 0.5 µm are melanin-containing vesicles that are specifically fabricate by melanocytes (23). As reported before, melanosome-miR-211 released by melanoma cells induced NFs to CAF transition upon absorbed by NFs, whereas the depletion of miR-211 prevented the formation of CAFs. Furthermore, miR-211 was found to target tumor suppressor IGF2R, resulting in the increase of collagen by CAFs so as to promote tumor cell motility (35). Although this study provided an opportunity to attenuate melanoma invasion by blocking the transformation of CAFs, whether melanosomes-miRNA can be absorbed by other cells in the melanoma microenvironment deserves more research. In addition, more efforts to explore EVs-miRNA mediated crosstalk between tumor cells and TME, as well as their potential to act as prognostic biomarkers or novel therapeutic strategy for melanoma should be considered. In addition to the transmission of tumor cell derived EVs to CAFs, EVs secreted by CAFs have also been revealed to exert an essential role in several tumor progression (24, 36). Recent data showed that CAF-released EVs enriched CD9 and CD63, and inhibited the proliferation of melanoma cells remarkably. The patients with CAF-derived CD9-positive EVs displayed a better five-year disease-free survival than patients with CD9-negative, indicating that CD9 expression in CAFs-EVs is a favorable prognostic marker for malignant melanoma patients (25). Furthermore, to develop novel targeted drugs depending on this type of EVs may be beneficial for the clinical treatment of melanoma patients. Moreover, tumor-derived EVs have been revealed to transdifferentiate CAFs by endothelial to mesenchymal transition (EndMT) pathway. By using an *in vitro* microfluidic model, which enables to observe the synergetic effect of TME in situ, Yeon et al. (37) found that the differentiated CAFs from human umbilical vein endothelial cells (HUVECs) were increased obviously when treated with melanoma-derived EVs, whereas melanoma-secreted EVs could promote EndMT. Predictably, this experimental model is expected to serve as a potent tool in the development of anti-tumor agents by exploring a variety of candidates, not just tumor derived EVs, which can depress the transformation of endothelial cells to CAFs.

**Table 1:** Crosstalk between EVs and stromal cells in melanoma.

Sources	Contents	Target	Biological Effect	Reference
Melanoma cells	Gm26809	Fibroblast	Reprogramming fibroblast cells to CAFs	(22)
Melanoma cells	miR-211	Fibroblast	Reprogramming fibroblast cells to CAFs	(23)
CAFs	CD9	Melanoma cells	Inhibiting the proliferation of melanoma cells	(24)
Melanoma cells	eTGF-β	HUVECs	Promote mesenchymal transition EndMT pathway	(25)
MSCs	miR-138-5p	Melanoma cells	Promoting the apoptosis of melanoma cells	(26)
MSCs	NEAT1	Melanoma cells	Inducing macrophages to M2 polarization	(27)
MSCs	miR-22-3p	Melanoma cells	Attenuating the EMT process of tumor epithelial cells	(28)

Apart from CAFs, mesenchymal stem cells (MSCs) is another essential stromal cells in the TME. EVs secreted by mesenchymal stem cells (MSCs) have shown huge potential for treating tumors because they can precisely locate TME (26). MSC-derived EVs was demonstrated to induce the apoptosis of melanoma cells via transmitting miR-138-5p, and then targeted SOX4. These results indicated the anti-tumor role of miR-138-5p/SOX4 axis during the malignancy of melanoma cells, and validated the potential therapeutic value of MSC-EVs based therapy for melanoma patients (27). In addition, Yang et al. (38) found that NEAT1 loaded in bone marrow mesenchymal stem cell-derived EVs could accelerate the progression of melanoma through inducing macrophages to M2 polarization, providing a novel target for the treatment of melanoma. In melanoma, epithelial-mesenchymal transition (EMT) is usually occurred when epithelial cells transit into mesenchymal cells, thereby promoting tumor metastasis and therapy resistance (28). Chen et al. (39) demonstrated that MSC-EVs carried miR-22-3p could reduce the expression of EMT related gene LGALS1 in melanoma cells so as to inhibit the EMT process of tumor epithelial cells. However, this study is lack of animal studies, and the therapeutic potential of MSC-EVs with miR-22-3p needed further investigation, especially the clinical trials.

## Melanoma-secreted EVs targeting angiogenesis

Angiogenesis is a biological process responsible for the formation of new blood vessels, thereby delivering oxygen and nutrients to tissues and organs in human body. Currently, angiogenesis has been considered to be a fundamental procedure in inducing benign tumors to malignant phenotype, such as invasion and metastasis (40). In TME studies, EVs released by tumor cells have been revealed to form the premetastatic niche by accelerating angiogenesis, indicating the vital role of EVs in the communication between tumor and angiogenesis (**Table 2**) (46, 47). It’s well known that the uPA/uPAR system components (urokinase-type plasminogen activator, uPA; uPA receptor, uPAR) are regarded as critical biomarkers for malignancy, including melanoma (41, 48). By utilizing subcutaneously implanted matrigel plugs containing EVs derived from wild type, uPAR- and uPAR+ melanoma cells, the group treated with uPAR+ EVs displayed more vascularized and micro-vessels, suggesting uPAR expressing melanoma EVs are crucial activators of angiogenesis (49). Therefore, depleting uPAR expression in tumor derived EVs is a promising method for treating melanoma, and the uPAR might be a useful biomarker when obtained EVs from melanoma patients by applying liquid biopsy. Hypoxia is a primary characteristic of solid tumors that is involved in tumor angiogenesis (50, 51). Notably, hypoxia also increases the secretion of EVs from tumor cells and alters the carriers of EVs (42). Tang et al. (52) found that hypoxic melanoma-derived small extracellular vesicles (sEVs) could enhance the angiogenic ability of CAFs by delivering the HSP90/p-IKKα/β complex to activate the IKK/IκB/NF-κB/CXCL1 signaling pathway in CAFs. Despite these finding offered a deeper understanding of the occurrence of angiogenesis in melanoma progression and provided promising targets, more clinical studies are necessary for estimating the therapeutic values of the HSP90/IKK enriched sEVs. The typical M2-like phenotype of TAMs can be induced by cytokines derived from Th2 cell, and IL-13 is the primary cytokine among them. Accordingly, targeting IL-13 receptor and ligand in the TME can block M2 polarization and then inhibit tumor growth (53). As reported, tumor cell-derived EVs are able to recruit and polarize TAMs to activate angiogenic signaling pathways via extensive molecule, including bFGF, TNF-α, and VEGF (43, 54). Given that, Negrea et al. (55) developed a novel nanosystem consists of Il-13-LCL-SIM (Il-13-conjugated long-circulating liposomes with SIM) to target TAMs and PEG-EV-DOX (PEG stabilized EVs with DOX) to target melanoma cells. In this study, it was shown that Il-13-LCL-SIM mainly impaired the pro-angiogenic functions of TAMs and reduced the expression of key proangiogenic proteins (such as VEGF), which further sensitized TME to the killing effects of PEG-EV-DOX. Therefore, combing EVs based therapy with other targeted strategies, including targeted angiogenesis, is promised to be an advantageous method to improve the efficacy of EVs-drugs in melanoma, which may also be applied in other malignant tumors.

**Table 2:** Communication between EVs and angiogenesis in melanoma.

Source	Contents	Target	Involved process	Reference
Melanoma cells	uPAR	HMVECs and ECFCs	Pro-angiogenic effects HMVECs and ECFCs	(41)
Melanoma cells	HSP90/p-IKKα/β complex	CAFs	Activating the IKK/IκB/NF-κB/CXCL1 axis in CAFs and promote angiogenesis *in vitro* and *in vivo*	(42)
Synthetic nanosystem	Il-13-LCL-SIM and PEG stabilized EVs with DOX	Melanoma cells	Il-13-LCL-SIM impaired the pro-angiogenic functions of TAMs and reduced the expression of VEGF, sensitizing TME to the killing effects of PEG-EV-DOX	(43)
Melanoma cells	IL-6, IL-8, VEGF and MMP2	Angiogenesis	Accelerating the angiogenesis processes and creating an immunosuppressive TME of melanoma	(44)
Melanoma cells	miR-155	CAFs	Triggering the proangiogenic switch of CAFs	(45)

Wnt signaling proteins are highly conserved proteins that are tightly involved in the developmental processes, and also multiple diseases, including cancers (56, 57). As an atypical Wnt signaling, WNT5A can promote the spread to distant tissues or organs and the formation of metastatic foci of melanoma, whereas the high expression of WNT5A commonly predicted a poor prognosis in melanoma patients (44, 58). In the endogenous WNT5A low expressed melanoma cells, the stimulation with rWNT5A led to a great release of EVs carrying the immunoregulatory cytokine IL-6, and more important the pro-angiogenic agents, including IL-8, VEGF and MMP2. In particular, these EVs might accelerate the angiogenesis processes and create an immunosuppressive TME of melanoma, thus inducing more rapid tumor growth and distinct metastasis (59). Consequently, targeting WNT5A thereby inhibiting the secretion of related EVs might be a promising strategy for the treatment of melanoma patients. However, more studies are needed to assess the potential side effect caused by the silence of WNT5A. Previous studies have proved that JAK2/STAT3 signaling pathway can regulate proangiogenic modulators expression, such as VEGFa, FGF2, and MMP9 (60, 61). Additionally, SOCS1 (suppressor of cytokine signaling 1) is a powerful inhibitor of JAK2/STAT3 signaling, however, SOCS1 is decreased in a great number of tumors and closely related to tumor angiogenesis (45, 62). Zhou et al. (63) demonstrated that melanoma cell-derived EVs could deliver miR-155 to fibroblasts NIH/3T3 and then elevated the expression of proangiogenic factors, including VEGFa, FGF2, and MMP9, by directly targeting SOCS1, thus triggering the proangiogenic switch of CAFs. Furthermore, *in vitro* and *in vivo* assays showed that treatment with EVs with overexpressed miR-155 promoted angiogenesis, whereas the knockdown of miR-155 in melanoma cell-released EVs mitigated angiogenesis obviously. This discovery may provide a new therapeutic target for anti-angiogenic therapy in the treatment of melanoma. Nonetheless, the inhibition of miR-155 in melanoma cell-derived EVs cannot diminish the proangiogenic regulators to the original expression level, combined with other therapies may be a feasible option to obtain better clinical benefits.

## EVs regulating immunoresponse in melanoma

**Figure 2:**
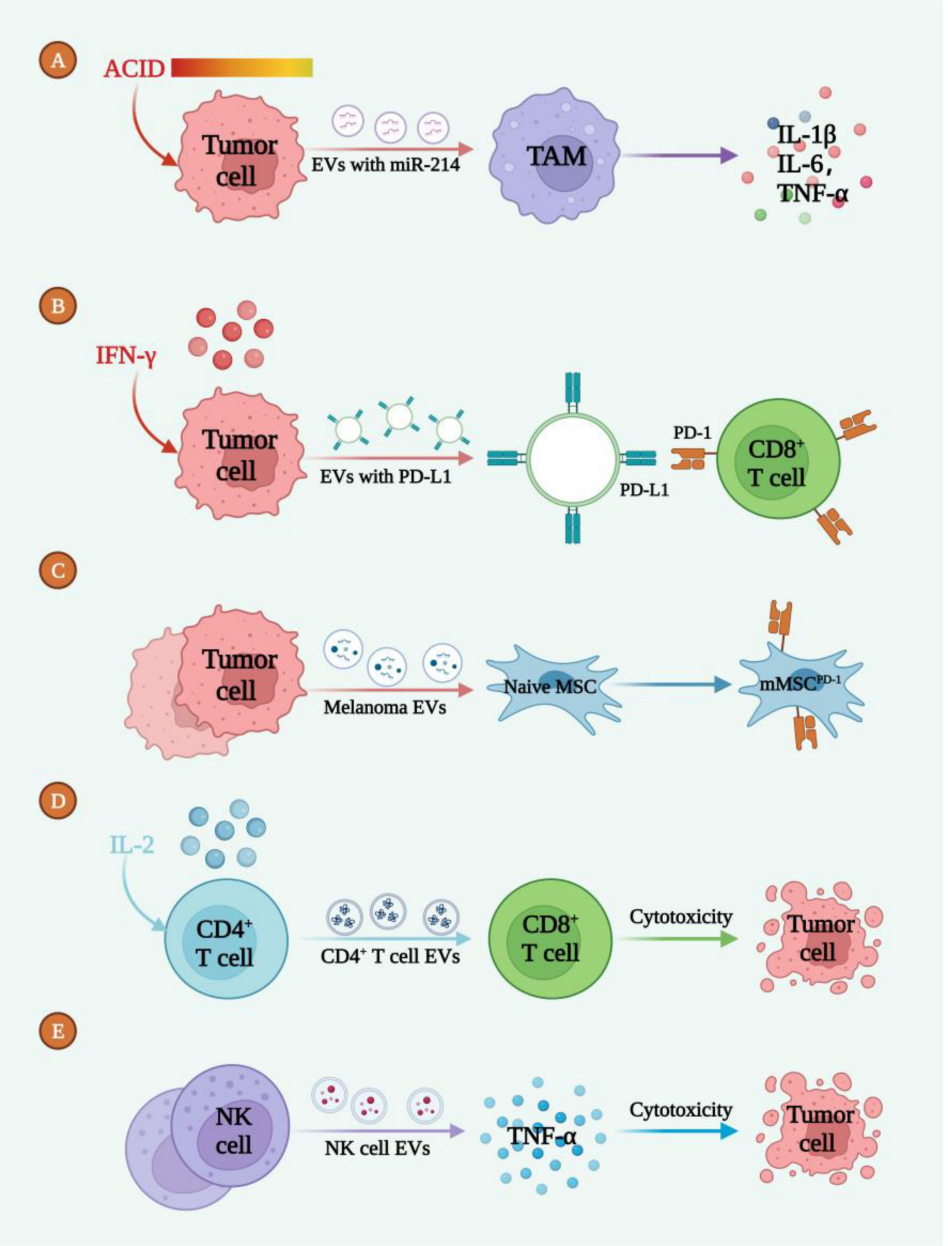
EVs mediated the intercommunication between melanoma cells and immunoresponse. **(A)** The acidic TME promoted the release of EVs-miR-214 to target TAM, inducing the inflammatory microenvironment. **(B)** IFN-γ induced tumor cell deriving EVs enriched with PD-L1 to bind with CD8+ T cell. **(C)** EVs secreted by melanoma cells induced the formation of mMSC^PD-1^. **(D)** IL-2 led to the release of CD4+ T cell EVs which caused tumor cell death. **(E)** NK-cell derived EVs secreted TNF-α to induce tumor cell death.

The TME is infiltrated by multiple immune cells, including lymphocytes (T cells, B cells, NK cells, and T regulatory cells), dendritic cells (DCs), tumor associated macrophages (TAMs), myeloid-derived suppressor cells (MDSC), as well as granulocytes (neutrophils, basophils, eosinophils, and mast cells). However, it has been recognized that tumor cells can regulate signaling pathways involved these immune cells and switch them to an immunorepressive manner, thus inducing enhanced tumor growth (64, 65). EVs comprise several tumor antigens which may result in the immunosuppression, yet increasing studies have indicated that tumor derived EVs are essential mediators between tumor cells and immune response (**Figure 2**) by releasing immune-associated factors to the TME (**Table 3**) (76, 77).

**Table 3:** EVs regulating immunoresponse in melanoma.

Related immunoresponse	Origin of EVs	Cargo	Target		Involved process	Reference
TAM	Melanoma cell	miR-214	COX-2		Inducing a proinflammatory activation of macrophages	(66)
	Panax ginseng C. A. Mey	DGMG, PE, Cer	TLR-4/MyD88 signaling pathway		Promoting M1-like polarization	(67)
ICBs	Melanoma cell	PD-L1	PD-1		Targeting PD-1+ CD8 T cells	(68)
	MSCs	Multiple upstream miRNAs and proteins	Activating tumorigenic signaling (e.g. PD-1, MET, RAF1, STAT3, BCL2, or mTOR		Formation of mMSC^PD-1+^	(69)
	BMDCs	MHC Class II and OVA antigen	antigen-specific CD8+ T cells		Inducing strong antigen specific T cell reaction	(70)
	Melanoma cell	HSP86	TLR4/NF-κB		Triggering the conversion of normal myeloid cells into MDSCs	(71)
	cell	PD-L1	PD-1		The exhaustion of tumor-specific CD8+ T cells	(72)
T cell	CD4+ T cell	miR-25-3p, miR-155-5p, miR- 215-5p, and miR-375	Perforin, granzyme B, and IFNγ		Increase of the cytotoxicity of CD8+T cell	(73)
NK cell	NK cell	NKG2D, CD94, CD40L	CD56+ NK cell fraction		Coordinating with NK-mediated immunosurveillance	(74)
	NK 92 cell	TNF-α	FasL		Inhibiting melanoma cells vibility	(75)

In addition, T regulatory cells, as immunosuppressive cells, inhibit the antitumor immune response and secrete various immunosuppressive cytokines, promoting the tumor immune escape (78). Melanoma cell derived EVs induce immune suppression by promoting T regulatory cell expansion as well as the demise of antitumor CD8+ effector T cells, thereby enabling tumor cell escape (79). Another study showed, the transfer of miRNA-214 from Melanoma derived EVs to T-cells down-regulates PTEN while favoring the expansion and migration of T regulatory cells in TME (80).

### EVs with TAM

The macrophages in TME are mainly divided into two categories: anti-tumor “M1” and tumor supportive “M2” types. In particular, M2 and partial M1 macrophages are considered as TAMs, which is the major immune population in TME and involved in tumor occurrence and development (66, 81, 82). A high TAM infiltration often leads to poor clinical outcomes in a great number of tumors, including melanoma (67, 83, 84). Besides, extracellular acidosis is another crucial aspect of the TME and Andreucci et al. (85) observed that the acidic microenvironment stimulated the secretion of melanoma-EVs enriched with miR-214. Then, these EVs tended to induce a proinflammatory activation of macrophages by increasing the expression of COX-2 and the release of inflammatory cytokines, such as IL-1β, IL-6, TNF-α, and NO, so as to establish an inflammatory TME which in turn facilitated the progression of melanoma. Hence, improving the acidic TME to reduce the release of miR-214-enriched EVs or diminishing the expression of miR-214 in melanoma derived EVs may be potential strategies for the treatment of melanoma. However, this study lacked primary macrophages experiments, and the polarization of macrophages was not detected, further research is urgent. Accumulating evidence showed that natural and modified EVs are capable of inducing a tumor suppressive response in macrophages to attenuate tumor growth (86, 87). From the roots of *Panax ginseng* C. A. Mey, nanoparticles similar to EVs was isolated and purified successfully, termed as ginseng-derived nanoparticles (GDNPs), which were enriched with digalactosyl monoacylglycerol (DGMG), phosphatidyl ethanolamine (PE), and ceramide (Cer) (88). When being internalized by TAM, GDNPs could promote M1-like polarization via TLR-4/MyD88 signaling pathway and increase the production of total ROS, which then induced the apoptosis of mouse melanoma cells and inhibited tumor growth *in vivo*. This work demonstrated that GDNPs played an immunoregulatory role on murine macrophages to suppress tumor growth *in vivo* and provided the valid foundation for further application as nanodrugs to treat melanoma, whereas more studies are needed to clarify the active ingredient in GDNPs, as well as the necessary clinical trials.

### EVs with immune checkpoint blockade

Notably, melanoma is a highly immunogenic malignancy characterized by the deeply lymphoid infiltration, thereby providing well foundation for the immunotherapy in melanoma (89, 90). Based on this, some immunotherapeutic strategies, especially the immune checkpoint blockade (ICB), have been demonstrated to be favorable for the refractory melanoma by activating effector T cells (68, 91). Compared with other immunotherapies, even in patients with advanced cancer, ICB therapies can usually illustrate higher response rates and persistent responses (69). Among them, PD-1/PD-L1 and CTLA-4/B7 immune checkpoint pathways are principal targets for ICB, which have been well studied in the past decades (92).

By using reverse phase protein array (RPPA) and western blot assay, Chen et al. (93) found that PD-L1 was significantly higher in EVs, mostly is exosomes, released from metastatic melanoma cells than that from primary melanoma cells. Mechanically, the abundance of PD-L1 on melanoma EVs is raised by IFN-γ, and EVs-PD-L1 chiefly targets PD-1^+^ CD8 T cells and promotes tumor growth *in vivo*. Moreover, in patients with advanced melanoma, the expression of circulating EVs-PD-L1 positively related to that of IFN-γ, and changes during the anti-PD-1 therapy with pembrolizumab. High levels of EVs-PD-L1 can reflect the exhaustion of T cells to the limit in melanoma patients, by which the T cells can no longer be re-activated by the anti-PD-1 treatment. Accordingly, to develop EVs-PD-L1 as a biomarker may be attractive. Recently, it has been found that melanoma cells with upregulated PD-1 are highly invasive (94), but it is still not clear how the PD-1 overexpressing subpopulations are generated. As reported, melanoma derived EVs could lead to the formation of a PD-1 overexpressing cell cluster (melanoma-like MSC^PD-1+^, mMSC^PD-1+^) from naive MSCs, by carrying a complex reprogramming of carcinogenic molecules (70). Furthermore, EVs and EVs activated mMSC^PD-1+^ cells are able to facilitate melanoma tumor progression *in vivo*, because of their highly expression of oncogenes and reduced susceptibility to programmed cell death, highlighting the complexity of EVs communication during the progression of melanoma. Although targeting PD-1/PD-L1 axis can mitigate T-cells exhaustion, it is not effective for all patients with cancer (95). This therapy resistance may be triggered by the deficient primary T-cells activation to tumor antigens, impaired antigen presentation, and decreased T-cell infiltration in the TME (96, 97). In view of this, increasing efforts have been made to explore more effective combination strategies. To investigate whether EVs related therapy could improve the tumor cells susceptibility to anti-PD-1/PD-L1 therapy in a checkpoint-resistant B16 melanoma model, Veerman et al. (98) injected bone marrow dendritic cell (BMDC)–derived EVs, but not checkpoint blockade, into tumor-bearing mice and then induced a strong antigen-specific T-cell response and impaired tumor growth. This demonstrates that the pretreatment with EVs can enhance anti-tumor immune responses in cancers refractory to checkpoint, and sensitize this subset of cancers to anti-PD-1/PD-L1 therapy. Therefore, EVs treatment has the great potential to collaborate with checkpoint blockade therapy, such as PD-1/PD-L1 blockage, in clinical application. Meanwhile, it is expected to be an alternative option for intractable tumors which are reactionless to checkpoint blockade. Myeloid-derived suppressor cells (MDSC) have been confirmed to be a critical role in the TME, and its accumulation in preclinical melanoma mouse models and melanoma patients can promote melanoma tumor progression via inhibiting T and NK cells (71, 72). The mechanism of MDSC-mediated immunosuppression is mainly related to the upregulation of PD-L1 binding with PD-1 expressed on the surface of tumor-infiltrating T cells (99). It has been proved that MDSC could also be derived from immature myeloid cells (IMC) or differentiated myeloid cells by the exposure to EVs released by tumor cells (100). Fleming et al. (101) highlighted that melanoma-derived EVs triggered the conversion of normal myeloid cells into MDSCs by the inducible HSP86 in EVs, which activates TLR4 on myelocytes and lead to the activation of NF-κB as well as the upregulation of PD-L1 expression. Furthermore, the knockdown of HSP86 in melanoma cells prohibited the secreted EVs to upregulate PD-L1. Although it may be primarily owing to the silence of HSP86 on the surface of EVs, whether HSP86 is responsible for the sorting of a variety of compounds into melanoma-EVs requires further exploration. By establishing an experimental pulmonary metastasis model using melanoma cells, Chen et al. (102) discovered melanoma cell-released EVs enriched PD-L1 were responsible for the metastatic progression by inducing the exhaustion of tumor-specific CD8^+^ T cells, providing a potential target for the treatment of melanoma. However, how to inhibit the release of tumor derived EVs, or reduce the enrichment of PD-L1 into EVs is a great challenge to be resolved.

In addition, the assessment of PD-L1 in tumor tissues has provided a feasible method to identify a patient population sensitive to chemotherapy (103). Nevertheless, PD-L1 levels could be influenced by the activity change of some signaling transduction pathways (104), thus abating its predictive value, whereas EVs-PD-L1 level may be a more appropriate indicators (105, 106). In order to explore other potential biomarkers, a study enrolled in 18 melanoma patients was conducted to evaluate whether PD-L1 mRNA level in plasma-derived EVs could reflect response to the anti-PD-1 agents, such as nivolumab and pembrolizumab. The results showed that PD-L1 expression in plasma-derived EVs decreased obviously in subjects responding to treatment but increased in that with advanced disease, suggesting that PD-L1 expression level in plasma-derived EVs may be a stable and valuable biomarker for the prediction of melanoma patient response to anti-PD-1 antibodies (107). However, it is necessary to collect a large number of clinical samples for further validation. Apart from that, Serrat et al. (108) identified the level of circulating PD-L1^+^ EVs released from melanoma and CD8^+^ T cells and that of PD1^+^ EVs were much higher in unresponsive patients. Besides, the Kaplan-Meier curves showed the higher levels of PD1^+^ EVs were significantly correlated with poor prognosis. This finding provides the probability for utilizing these circulating EVs, which might be employed as low invasive liquid biopsy to monitor the response of melanoma patients to ICB therapy.

### EVs derived from immune cells

In the TME, immune cells released EVs have attracted much attention for cancer immunotherapy, because they also display immunological endogenous features similar to their parental cells (73, 109). Among them, T cell-derived EVs have been reported to exert anti-tumor effects in cancer immunotherapy through mimicking their parental cells (110, 111). As reported, IL2-stimulated CD4^+^ T cell-derived EVs played certain roles in anti-tumor immune responses via increasing the cytotoxicity of CD8^+^T cell. Furthermore, in a tumor-bearing mouse model, it was demonstrated that EVs secreted by CD4^+^ T cell attenuated the growth of melanoma significantly via CD8^+^ T cell mediated tumor suppression (112). Consequently, EVs originated from CD4^+^ T cells are promising therapeutic agents to induce potent anti-tumor responses for melanoma patients.

NK cells consist of many granular lymphocytes, and play a leading role in the anti-tumor immune responses by inhibiting tumor growth, and metastasis (74). In the immune microenvironment, NK cells communicate with other immune cells, such as DCs, T and B cells, to modulate innate and adaptive immune responses. This crosstalk is mediated by the release of a wide range of modulators, including cytokines, chemokines, and EVs (75, 113). In particular, NK-cell-derived EVs (NKEVs) are constitutively released and have anti-tumor activities similar to NK cells (114). Moreover, in both NK-derived exosomes and microvesicles, NKEV mass spectrometry and cytokine profile identified the expression of NK cell markers, such as NKG2D, CD94, CD40L, and other cytotoxic molecules, as well as the factors involved in cell adhesion, and immune response, suggesting that NKEVs are potential agents to be applied in cancer therapy. In melanoma patients, it was demonstrated that the quantity of circulating NKEVs is much lower than that in healthy donors, which might result in the low immune activity in melanoma patients. Hence, NKEVs could act as collaborator to coordinate with NK-mediated immunosurveillance, and the supplement of normal NKEVs to enhance the efficacy of immunotherapies might be a favorable option for melanoma patients (115). In addition, after being co-cultured with NK-92 EVs, the melanoma cells exhibited low cell viability but high cell apoptosis in a does dependent manner, which might be mediated by the secretion of TNF-α from NK-92 EVs (116). The inhomogeneity of the TME, especially the acidity distributed in the TME causes the reduction of perforin/granzymes from NK cells and inhibition of Fas/FasL interaction. However, acidity is able to promote the accumulation and release of EVs due to the low pH of TME attracts them and induces membrane fusion (117). Hereby, NK-92 EVs based immunotherapy has obvious advantages over therapy dependent on whole NK cell, and deserves further utilization as a promising immunotherapeutic strategy for melanoma.

## EVs remodeling ECM in melanoma

**Figure 3:**
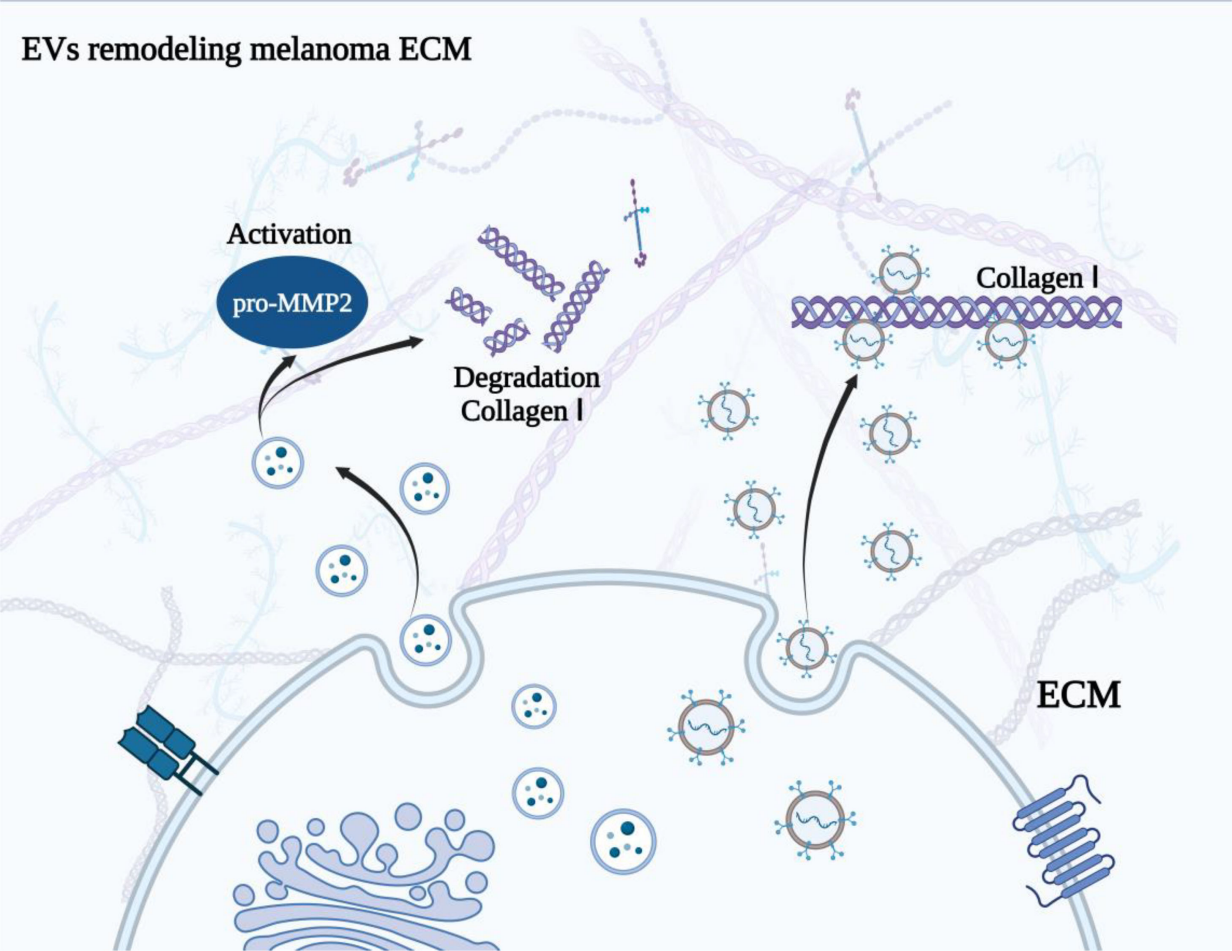
Melanoma derived EVs remodel ECM. The physical interaction between EVs and collagen I enhanced the activity of metalloproteases at the surface of EVs and the remodeling of the ECM. Besides, EVs with MT1-MMP could activate pro-MMP-2 and degrade type 1 collagen, resulting the remodeling of ECM.

Extracellular matrix (ECM) is a major non-cellular component in TME (118). It has been well demonstrated that ECM is a macromolecular network comprising collagens, elastin, fibronectin, proteoglycans/glycosaminoglycans, and laminins, which acts as a physical scaffold and is exerts essential roles in tumor cell differentiation, migration, and homeostasis (119, 120). The remodeling of ECM arises under physiological and pathological conditions and is regulated by multiple enzymes, such as metalloproteinases. Some EVs have been observed within the matrix and involved in the remodeling of ECM, that will impel the modification of the TME, and the formation of pre-metastatic niches (121, 122). Palmulli et al. (123) found that melanoma-derived sEVs are able to physically interact with collagen I, the primary fibrous component of the ECM, and then enhanced the activity of metalloproteases at the surface of EVs and the remodeling of the ECM, which is involved in the advancement of malignant tumors. Accordingly, blocking the binding between tumor derived EVs with collagen I may be a feasible therapeutic strategy for melanoma patients (**Figure 3**). However, whether EVs are capable of binding to other collagens or other components of ECM to remodel TME is still a challenge. Matrix metalloproteinases (MMPs) are zinc-dependent proteins, which take part in the tissue remodeling and involve tumor progression (124, 125). The membrane type 1 matrix metalloproteinase (MT1-MMP) is a critical member of metalloproteinases that facilitate tumor progression by remodeling the ECM (126, 127). From the culture medium of melanoma (G361) cells, EVs carrying the full-length and the proteolytically processed forms of MT1-MMP were isolated and identified (128). Interestingly, the EVs could activate pro-MMP-2 and degrade type 1 collagen, indicating EVs-MT1-MMP possessed functional activities. This provided a novel mechanism by which melanoma cells could remodel the ECM in TME, thus targeting MT1-MMP in EVs, or depressing the release of tumor cell derived EVs might be a promising strategy for melanoma treatment.

## Conclusions and perspective

Owing to the essential roles of TME in the occurrence and development of tumors, including melanoma, targeting of the TME has recently been regarded as a great promising therapeutic strategy to improve the anti-cancer efficacy (129, 130). Along with the clinical approval of several drugs and targeted therapies that were designed toward the angiogenesis, immune checkpoints, T and NK cells, additional molecules directly target the cellular or non-cellular components of TME are also under development. In this review, we have highlighted the potential of EVs mediated the crosstalk within the TME to be the therapeutic agents for melanoma patients. Moreover, emerging studies have also revealed that plasma-derived EVs can act as biomarkers for the advancement of melanoma, such as the circulating EVs-PD-1/PD-L1, thus providing a novel direction for the lipid biopsy. Although EVs-related therapies and biomarkers have shown huge application prospects, some shortages still needed to be overcome before its clinical use. For example, the heterogeneity in size, composition and sources of EVs lead to the difficulty to isolate a specific subset with desirable factors, which will reduce the accuracy of targeting therapy and bring some unexpected side effects. Therefore, the isolation and characterization methods should be further optimized to obtain more ‘pure’ EVs. Besides, the natural EVs commonly face problems such as the low production and complex composition. Furthermore, the efficiency of EVs may also be declined due to the low infiltration, insufficient drug delivery, captured by immune cells, and the inadequate immune response (131–133). Recently, a number of engineering methods have been used to obtain generous artificial and modified EVs to improve EVs efficacy and practicability, which have shown encouraging results.

In addition to carry endogenous cargoes, because of the inherent protection of the cargo and capable of enhancing solubility, stability, and specificity, EVs have been proposed as a drug delivery system to load single or multiple drugs to exert anti-tumor effect. In the clinical treatment of melanoma, ICBs are widely used. Currently, several monoclonal antibodies, such as pembrolizumab and nivolumab (anti-PD-1), and ipilimumab (anti-CTLA-4) have been studied and become the appropriate therapy to improve metastatic melanoma survival (134–138). However, there are still around 50% of patients fail to achieve clinical benefits (139). Utilizing EVs to delivery these antibodies to the TME, thus directly targeting tumor cells or T cells may be an alternative method to improve the efficacy of ICBs in melanoma. Moreover, modifying EVs to enhance their melanoma TME targeting will be favorable for increasing the accumulation and sustain of ICIs in the TME. However, few engineered EVs loaded with ICIs or other anti-tumor modulators specially targeting melanoma has been developed, whereas more efforts are needed.

Taken together, although the therapeutic application of EVs in melanoma clinical treatment is still restricted by the deficiency of feasible and standardized methods to obtain moderate EVs from melanoma patients biofluids and tumor cells, the limited loading efficiency of multiple agents, and the difficulty to manufacture sufficient melanoma specific EVs, EVs are still the great promising therapeutic manner for melanoma. With the progress of technology and new findings, EVs will finally shed light on the melanoma diagnosis and treatment in the era of precision medicine.

## Author contributions

YL: Conceptualization, Methodology, Software Data curation, Writing- Original draft preparation. FL: Visualization, Investigation, Supervision. Writing- Reviewing and Editing. All authors have read and agreed to the published version of the manuscript.
